# Assessment of Quality and Environmental Impact of Artisanal Fresh Pasta Fortified with Agri-Food By-Products

**DOI:** 10.3390/foods14193379

**Published:** 2025-09-29

**Authors:** Manazza Ayub, Alessia Le Rose, Olimpia Panza, Dario Caro, Matteo Alessandro Del Nobile, Amalia Conte

**Affiliations:** 1 Department of Economics, Management and Territory, University of Foggia, Via A. da Zara, 71122 Foggia, Italy; manazza.ayub@unifg.it; 2 Ecodynamics Group, Department of Physical Sciences, Earth, Environment, University of Siena, Piazzetta Enzo Tiezzi, 1, 53100 Siena, Italy; alessia.lerose@student.unisi.it (A.L.R.); caro2@unisi.it (D.C.); 3 Department of Science, Technology and Society, University School for Advanced Studies IUSS Pavia, 27100 Pavia, Italy; 4 Department of Humanistic Studies, Letters, Cultural Heritage, Educational Sciences, University of Foggia, Via Arpi, 71121 Foggia, Italy; olimpia.panza@unifg.it (O.P.); amalia.conte@unifg.it (A.C.)

**Keywords:** fresh pasta, olive pomace, artichoke by-products, fortification, sustainability

## Abstract

Fresh pasta was enriched with olive pomace (OP) and artichoke by-products (AB), respectively, at three concentrations: 13.5%, 14.5%, and 15% for OP, and 15%, 17%, and 19% for AB. Both control and fortified samples were assessed for technological properties, nutritional content and sensory quality. A Life Cycle Assessment was also performed to estimate the carbon footprint associated with pasta production. Results demonstrated a worsening of pasta quality, above all the resistance to break for row pasta and sandiness and taste for cooked samples, even though the pasta remained in an acceptable range. Fibers, polyphenol content, flavonoids, and antioxidant activity were found to be better in fortified samples than in the control pasta. With AB as new ingredient, the antioxidant activity increased substantially. The environmental impact revealed two different scenarios: compared to the control (1.08 kgCO_2_eq), lower carbon footprint values were found for pasta fortified with OP (from 0.96 to 0.98 kgCO_2_eq) and higher values for pasta fortified with AB (from 1.53 to 1.62 kgCO_2_eq), due to the energy consumption associated with by-product processing (dehydration at 50 °C and grinding). Thus, combining sensory quality, nutritional improvements and environmental impact, a Global Quality Index (GQI) was also calculated for each sample. The GQI values, according to the weighting scheme of this index, revealed that the benefits of AB superimposed the drawbacks and suggested that 15% AB fortification is the best solution to balance pros and cons of by-product recycling.

## 1. Introduction

Over recent decades, rapid increase in the global population and advancements in agri-food industrial processing have drawn attention towards sustainability and minimizing food waste, especially through the valorization of food by-products. The total cost of global food waste has reached $2.5 trillion per year, leading to more environmental concerns and a need for advanced food waste management technologies [1]. Simultaneously, consumer demand for healthier and functional foods has grown significantly, generating interest in incorporating by-products into traditional foods [2,3]. In general, by-products such as peels, seeds, stems, leaves, and other industrial residues are frequently discarded in landfills or incinerated, causing environmental and health hazards, along with considerable loss of nutrient-rich materials [4,5]. As a result, these impacts have prompted the food industry to reutilize by-products within the food chain, enriching food products with bioactive compounds, while addressing environmental, economic, and health concerns [6,7].

Artichoke (*Cynara scolymus*), belonging to the family Asteraceae, widespread in the Mediterranean region, is well known for its important role in human diet, making it a valuable nutritional vegetable [8]. In 2023, worldwide production of artichokes averaged 1,609,935.39 tons, with Egypt, Italy, and Spain as the leading cultivators [9]. The edible parts of artichoke are immature inflorescences (capitula), internal fleshy leaves (bracts), and the heart (receptacle), which can be consumed in raw, cooked, roasted, baked, or canned form, while the inedible parts are the roots, stem and outer bracts [10]. Industrial processing of artichoke produces large quantities of by-products as the edible portion of artichoke just consists of 15–25% of the total fresh weight, whereas the inedible portion comprises about 75–85% of the total fresh weight [11]. These by-products, if discarded as food waste, can adversely affect the environment. However, due to their rich nutritional profile, they are of considerable interest to various industries, including agricultural, pharmaceutical, paper and pulp bioplastics, biomass and bioenergy, and cosmetic and food industries. Artichoke by-products are rich in phenolic compounds (caffeic acid derivatives), flavonoids (e.g., luteolin and apigenin, cyanidin caffeoyl glucoside derivatives), and dietary fiber, both soluble and insoluble. The insoluble fiber is mainly constituted of cellulose and hemicellulose, while the soluble fiber fraction is mainly formed by inulin, which is characterized by its prebiotic and bifidogenic effects [12]. The bioactive compounds confer the potential to work as hepatoprotective, choleretic, cardio-protective, anticarcinogenic, antispasmodic, anti-hypercholesterolemic, anti-inflammatory, antiviral (anti-HIV), antibacterial, and antioxidant agents [13], even though drying treatments and process conditions can affect their stability [14,15]. In the food industry, artichoke by-products have found various applications, including the use as a preservative in canola oil and tomato juice [16,17], emulsifying agent in oil microencapsulation [18], vegetable coagulant in cheese production [19], and natural source of dietary fiber and antioxidants in a variety of food products [20,21].

Olive oil processing generates significant amounts of waste materials, one of these being the olive pomace. This is a solid by-product, consisting of crushed olive stones, pulp, skin, and water [22]. Composition and amount of olive pomace can vary depending on the cultivar and olive oil processing technique. Pressing 1000 kg of olives approximately yields between 550 and 800 kg of olive pomace, with the exact amount depending on the oil extraction method used. The disposal of this pomace presents environmental challenges [23]. Olive pomace is a good source of polysaccharides (cellulose, hemicellulose, pectin), tocopherols, sterols, hydrocarbons, fatty acids, minerals, phenolic polymers (lignin), and polyphenols (particularly hydroxytyrosol and tyrosol derivatives, seco-iridoids, flavonoids, and phenolic acids) [24]. Olive pomace has been used in food preservation and food fortification. It has been added in edible oils [25], bakery products (bread, biscuits and snacks) [26], pasta [27], milk [28], and meat [29,30] to either improve their nutritional value or prolong the shelf life.

In light of these considerations, the current research aimed to analyze the potential of incorporating olive pomace and artichoke by-products into artisanal fresh pasta formulations by assessing their impact on energy consumption during dehydration and grinding, as well as on sensory, technological, and nutritional properties. The novelty lies precisely in the valorization of residual horticultural matrices to verify the real convenience in transforming these environmental burdens into valuable resources for the functional enrichment of fresh pasta. The use of food by-products as innovative ingredients can improve resource use efficiency and contribute to the transition towards a circular economy [31]. Building on this, the study further seeks to compare the overall quality of these formulations based on two key aspects, environmental sustainability (in terms of carbon footprint) and nutritional enhancement, to determine the most feasible approach for functional and eco-friendly fresh pasta production.

## 2. Materials and Methods

### 2.1. Raw Material Processing and Dehydration

Artichoke by-products (AB) with an initial moisture content of approximately 47% were obtained from a local farm in Foggia (Italy), and the olive pomace (OP) with an initial moisture content of approximately 85% was obtained from an olive mill in South Italy. The artichokes were thoroughly washed with tap water, disinfected by immersion in a 20 mL L^−1^ chlorinated water for 5 min, and rinsed again with tap water. Artichoke by-products were manually separated using a knife and cut into uniformly sized pieces. Both the artichoke and olive by-products were stored at −18 °C to prevent microbial contamination prior to processing. Before dehydration, samples were defrosted under refrigeration conditions. Dehydration was carried out in a conventional hot air dryer (PF-SIC CO80PRO, SICCOTECH, Campobasso, Italy) at 50 °C and 5% relative humidity. About 6 kg of fresh by-products were distributed in the cabinet (about 0.6 m^3^) over the racks in the form of uniform thin layers and dried until a constant moisture content was achieved. The air-drying process used forced convection at atmospheric pressure [32]. The dried materials were then ground using a lab grinder (KMEC Engineering, Kate Road, Anqiu City, Shandong province, China). Granulometric analysis was performed using a set of metal sieves (Enco S.r.l., Spinea, Venice, Italy) to determine the particle size distribution. The ground powders were further sieved using a 400 µm mesh sieve and stored in plastic bags at 4 °C until further use.

### 2.2. Pasta Production

Control pasta (CTRL) was prepared using durum wheat semolina, distilled water, and fresh eggs. Fortified pasta was prepared using durum wheat semolina, distilled water, fresh eggs, carboxymethylcellulose (CMC), by-products, and additional water to hydrate the by-products. CMC (SapurePuro, Gioia Group S.r.l., Turin, Italy) is a sodium salt, chemically derived from natural plant fibers of cellulose with degree of substitution 0.85-0.92; viscosity 1900–2600 mPa*s; sulfate ash 20–33.3%, sodium content 6.5–9.5%, sodium chloride content max 0.40%, and moisture 10%. It was used in new pasta samples at 2% *w*/*w* because it is well recognized that at this level it improves the texture and cooking quality of fortified pasta [33]. Three formulations were prepared for each by-product: OP_Low (13.5%), OP_Med (14.5%), and OP_High (15%) for olive pomace-fortified pasta, and AB_Low (15%), AB_Med (17%), and AB_High (19%) for artichoke by-product-fortified pasta. The amounts of semolina, distilled water, and fresh eggs remained the same in all the fortified pasta formulations as in the CTRL, but the quantities of by-products, CMC, and water added to hydrate the by-products varied, as shown in Table 1. These formulations came from a preliminary optimization process based on sensory evaluation. Several amounts of both OP and AB, and different hydration levels of by-products were tested to define the three concentrations able to fortify pasta without compromising the acceptability after cooking (score > 5). Pasta dough was prepared by uniformly mixing the ingredients in a pasta maker (Monferrina P3, Lineapasta, Veneto, Italy), for about 7 min. Then, the dough was extruded from the machine using a bronze die to form pasta in the form of *troccoli*.

### 2.3. Sensory Analysis

Samples were served to the panel consisting of ten well-trained members with several years of experience in pasta evaluation, as Researchers and PhD students of the Food Science Department of the University of Foggia. Fresh pasta was subjected to a quantitative descriptive analysis (QDA). The panel members, even if expert in pasta evaluation, were re-trained in a session (1 h/day for 2 days) to align their judgments and define the sensory parameters. For the test, 50 g pasta samples from each formulation were cooked at 100 °C in 500 mL of water until reaching the optimum cooking time (OCT) and then subjected to sensory analysis. Samples were presented at random for the assessment of the following attributes: color, odor, appearance, homogeneity, breaking strength, and overall quality of the uncooked pasta; adhesiveness, bulkiness, sandiness, firmness, elasticity, color, odor, taste, and overall quality of the cooked pasta. A nine-point scale was used for the sensory evaluation, where 1, 9, and 5 corresponded to the lowest score, the highest score, and the threshold of acceptability for each attribute, respectively. Each panelist used the same nine-point scale to assess the overall quality of both uncooked and cooked pasta samples. The experiment did not require any Ethics Committee approval because there were no associated risks for panelists. The samples were prepared according to good manufacturing practices. For protecting the rights and privacy of the participants to the panel, an appropriate protocol was adopted to consider the verbal consent of the participants, no coercion to participate, the ability to withdraw from the study at any time, the full disclosure of study requirements and risks, and not releasing participant data without their knowledge.

### 2.4. Technological Analyses

Technological properties such as optimal cooking time (OCT), cooking loss (CL), swelling index (SI), and water absorption index (WAI) were determined according to the AACC approved method 66-50 [34]. OCT was monitored by pressing pasta strands between two glass plates and observing the disappearance of the starchy central core at each 30 s interval during cooking. The time at which the central core fully disappeared was considered as the OCT. CL (%) refers to the solid material lost in the water used for cooking, measured by cooking 10 g of the raw sample in 300 mL of boiling distilled water until the OCT was reached. Cooking water was then collected in an aluminum container and dried in an oven at 105 °C until a constant weight was obtained. Residue was measured and reported as CL of the initial sample. To measure SI, raw sample was cut into pieces of equal length (4 cm), weighed (10 g), cooked, and oven dried at 105 °C until a constant weight was achieved. SI was expressed as follows: [(cooked sample weight − sample weight after drying)/sample weight after drying]. WAI (%) of 10 g of cooked and drained sample was calculated using the following formula: [(cooked sample weight − raw sample weight)/uncooked sample weight] × 100. Each measurement was taken in triplicate.

### 2.5. Chemical Analyses

The chemicals used for the analyses were the following: Folin–Ciocalteu reagent, gallic acid mono hydrate, Trolox (6-hydroxy-2,5,7,8-tetramethylchroman-2 carboxylic acid), 2,2-azino-bis (3-ethylbenzothiazoline-6-sulfonic acid), diammonium salt (ABTS), potassium persulfate, sodium nitrite, sodium hydroxide solution, and quercetin, methanol, supplied from Sigma-Aldrich (Milan, Italy). Anhydrous sodium carbonate was supplied from Carlo Erba (Milan, Italy). All the reagents were of analytical grade. To determine the total phenol content (TPC), total flavonoids (TFC), and antioxidant activity (ABTS) of original by-products and of fresh pasta samples, the extraction was performed as described by Panza et al. [35] with slight modifications. Initially, the *troccoli* were dried with a ventilated stove (BINDER GmbH, Tuttlingen, Germany) at 35 °C for 12 h and milled to obtain powder samples. The mixtures, 2 g of dry sample with 20 mL of methanol aqueous solution (80:20), were included in 50 mL centrifuge tubes, homogenized, and subjected to ultrasound treatment for 15 min, according to Natrella et al. [36]. Then, each extract was centrifuged at 10,000 rpm, at 4 °C for 15 min to recover the supernatant, which was collected and filtered (PTFE, 0.45 μm) and used for TPC, TFC, and ABTS assay. Three extractions were carried out for each sample. As described by Panza et al. [35], the total phenolic compounds (TPC) were determined by the Folin–Ciocalteu colorimetric method. TPC was expressed as mg of gallic acid (GAE)/g of dry weight (dw), according to a calibration curve (6.25–300 mg/L; R2 = 0.998). The total flavonoids (TFC) were determined by the aluminum chloride colorimetric method [35]. TFC was expressed as mg of quercetin equivalents (QE)/g dw, according to a calibration curve (25–800 mg/L; R2 = 0.996). The evaluation of antioxidant activity was performed with ABTS assay, as described by Re et al. [37]. ABTS method was expressed as mg of Trolox equivalents (TE)/g dw, according to a calibration curve (3.25–600; R2 = 0.992). Triplicate measurements were made for each sample.

### 2.6. Fiber Content

Both raw materials and pasta samples were analyzed for dietary fiber using the AOAC Official Method [38] and expressed in g/100 g. The analysis was conducted at the NIRO SRL laboratory in Campobasso (CB), Italy.

### 2.7. Energy Consumption for the By-Product Dehydration Process

The procedure used by Le Rose et al. [39] to determine the amount of energy consumed per gram of dehydrating by-product ($\tilde{E}\left(t\right)$) was used in this work. The by-product water content was evaluated according to the following equation:

(1) $$C\left(t\right)\;=\;\frac{W\left(t\right)-\;W_{F}}{W_{F}}\cdot 100$$

where $C\left(t\right)$ is the sample water content at time t expressed as $\left[\frac{g\;water}{100\;g\;dry\;matter}\right]$, $W\left(t\right)$ is the weight of sample at time t expressed as $\left[g\right]$, and $W_{F}$ is the weight of the sample after it has been kept at 130 °C until all the water was desorbed; it was expressed as $\left[g\right]$.

The amount of water desorbed at time t $\left(M_{H_{2}O}\left(t\right)\right)$ was calculated according to the following equation:

(2) $$M_{H_{2}O}\left(t\right)=\;C_{0}\;-\;C\left(t\right)$$

where $C_{0}$ is the initial sample water content. The amount of water desorbed at equilibrium $\left(M_{H_{2}O}^{\infty }\right)$ was estimated according to the following expression:

(3) $$M_{H_{2}O}^{\infty }=\;C_{0}\;-\;C_{\infty }$$

where $C_{\infty }$ is the sample water content at the end of the dehydration.

The by-product dehydration kinetic was described using the following relationships:

(4) $$0\;\leq \;t\;\leq \;t_{c}\;M_{H_{2}O}\left(t\right)=\;K_{1}\cdot t$$

(5) $$t>t_{c}\;M_{H_{2}O}\left(t\right)=K_{1}\cdot t_{c}+K_{2}\cdot \left\{1-exp\left[-\left(t-t_{c}\right)\cdot \left(\frac{K_{1}}{K_{2}}\right)\right]\right\}$$

where $K_{1}$ is the desorption rate during the 1st Stage $\left[\frac{g\;desorbed\;water}{100\;g\;dry\;matter}\cdot \frac{1}{min}\right]$, $K_{2}$ is maximum amount of water desorbed during the 2nd Stage $\left[\frac{g\;desorbed\;water}{100\;g\;dry\;matter}\right]$, $t_{c}$ is the moment in which the transition from one stage to another takes place, and t is the dehydration time. The reader is invited to refer to the study of Conte et al. [32] for a detailed description of the model used to describe the dehydration kinetic and the hypotheses used to derive it.

The amount of energy consumed per gram of dehydrating by-product is related to the extent of the dehydration process through the following expressions:

(6) $$0\;\leq \;ext\%\;\leq \;\frac{K_{1}\cdot t_{c}}{M_{H_{2}O}^{\infty }}\cdot 100\;\tilde{E}\left(ext\%\right)\;=\;\frac{\gamma \cdot \left(\frac{M_{H_{2}O}^{\infty }}{K_{1}\cdot 100}\cdot ext\%\right)}{m_{Tot}^{0}\cdot \left(1\;-\;\frac{x_{dm}^{0}}{100}\cdot ext\%\cdot \frac{M_{H_{2}O}^{\infty }}{100}\right)}$$

(7) $$ext\%>\frac{K_{1}\cdot t_{c}}{M_{H_{2}O}^{\infty }}\cdot 100\;\tilde{E}\left(ext\%\right)=\frac{\gamma \cdot \left[t_{c}-ln\left(\frac{K_{1}\cdot t_{c}\;+\;K_{2}\;-\;ext\%\cdot \frac{M_{H_{2}O}^{\infty }}{100}}{K_{2}}\right)\cdot \frac{K_{2}}{K_{1}}\right]}{m_{Tot}^{0}\cdot \left(1-\frac{x_{dm}^{0}}{100}\cdot ext\%\cdot \frac{M_{H_{2}O}^{\infty }}{100}\right)}$$

where γ is the energy power provided to the dehydrator, ext% is the extent of the dehydration process (i.e., $\frac{M_{H_{2}O}\left(t\right)}{M_{H_{2}O}^{\infty }}\cdot 100$), $m_{Tot}^{0}$ is the initial value of the mass of dehydrating by-product, and $x_{dm}^{0}$ is the initial value of dry matter mass fraction. The reader is invited to refer to the study of Le Rose et al. [39] for a detailed description of the procedure used in this work to determine the amount of energy consumed per gram of dehydrated by-product.

The time needed to reach a given extent of the dehydration process (i.e., $t_{ext\%}$) is related to ext% through the following expressions:

(8) $$0\;\leq \;ext\%\;\leq \;\frac{K_{1}\cdot t_{c}}{M_{H_{2}O}^{\infty }}\cdot 100\;t_{ext\%}\;=\;\frac{M_{H_{2}O}^{\infty }}{K_{1}\cdot 100}\cdot ext\%$$

(9) $$ext\%>\frac{K_{1}\cdot t_{c}}{M_{H_{2}O}^{\infty }}\cdot 100\;t_{ext\%}=t_{c}-ln\left(\frac{K_{1}\cdot t_{c}+K_{2}-ext\%\cdot \frac{M_{H_{2}O}^{\infty }}{100}}{K_{2}}\right)\cdot \frac{K_{2}}{K_{1}}$$

The reader is invited to refer to the study of Panza et al. [40] for a detailed description of the procedure used in this work to determine the time needed to reach a given extent of the dehydration process.

### 2.8. Environmental Impact Measurement

The Life Cycle Assessment (LCA) methodology adopted in this study follows the ISO 14040 [41] and ISO 14044 [42] standards, which outline the four main phases of a comprehensive assessment: defining the goal and scope, conducting inventory analysis, assessing the environmental impacts, and interpreting the results. The by-products used in the various pasta samples were considered generic biowaste and the associated carbon footprint was excluded from the analysis, meaning they were defined as “burden free”. The functional unit (FU) is a quantified description of the function provided by a product or system and is used as a reference for all assessments in the impact analysis [43]. In this study, the FU was defined as 1 kg of pasta. Except for the by-products used in the pasta production, the entire supply chain was assessed for all other ingredients. The environmental impact data for semolina were sourced from the Environmental Product Declaration (EPD) of “Pasta di Semola Agnesi” [44], as they were not available in the consulted databases. Furthermore, since the ingredient “egg” was not available in the used database, environmental data were sourced from Leinonen et al. [45] and Guillaume et al. [46]; both reported an identical carbon footprint value per kilogram of product. As distilled water used in the pasta preparation process is not available in the reference Life Cycle Inventory database, it was substituted with “tap water” [47]. In the Life Cycle Inventory (LCI) stage, the recycling process was assessed based on primary data, acquired through direct measurement during experimental activities conducted at the University of Foggia. Particular attention was given to quantifying the energy consumption associated with drying and milling of the by-products. During the Life Cycle Impact Assessment (LCIA) phase, a quantitative evaluation was carried out to assess the influence of the inputs and outputs from the inventory phase on the selected environmental impact category. This assessment used the “CML-IA baseline v. 3.06” method, applied through SimaPro software v. 9.6.0.1, which was used to estimate the potential impact within the targeted category. The environmental impact category selected for this study was the Global Warming Potential over 100 years (GWP100), expressed in kilograms of CO_2_ equivalent (kgCO_2_eq). Results associated with this impact category are commonly called “carbon footprint” [48].

### 2.9. Global Quality Index Calculation

The procedure used by Lordi et al. [49] was used to calculate the Global Quality Index (GQI). The positive aspects associated with fortification are related to both the reduced impact on the environment and the increase in nutritional quality (for example, Total Dietary Fiber and ABTS), while the negative aspect associated with fortification is Sensory Quality.

The normalization of the quality indices was made according to the following expressions:

(10) $$Normalized\;Environmental\;Impact=\;\frac{{PQI}_{EI}^{CTR}-\;{PQI}_{EI}^{Act}}{{PQI}_{EI}^{CTR}}\cdot 100$$

(11) $$Normalized\;Totale\;Dietary\;Fiber=\frac{{{PQI}_{TDF}^{Act}-PQI}_{TDF}^{CTR}}{{PQI}_{TDF}^{CTR}}\cdot 100$$

(12) $$Normalized\;ABTS=\frac{{{PQI}_{ABTS}^{Act}-PQI}_{ABTS}^{CTR}}{{PQI}_{ABTS}^{CTR}}\cdot 100$$

(13) $$Normalized\;Sensory\;Quality=\frac{{NQI}_{SQ}^{CTR}-{NQI}_{SQ}^{Act}}{{NQI}_{SQ}^{CTR}}\cdot 100$$

where ${PQI}_{EI}^{CTR}$ is the Quality Index of the control sample related to the Environmental Impact, ${PQI}_{EI}^{Act}$ is the Quality Index of the active sample related to the Environmental Impact, ${PQI}_{TDF}^{CTR}$ is the Quality Index of the control sample related to the Total Dietary Fiber, ${PQI}_{TDF}^{Act}$ is the Quality Index of the active sample related to the Total Dietary Fiber, ${PQI}_{ABTS}^{CTR}$ is the Quality Index of the control sample related to ABTS, ${PQI}_{ABTS}^{Act}$ is the Quality Index of the active sample related to ABTS, ${NQI}_{SQ}^{CTR}$ is the Quality Index of the control sample related to the Sensory Quality, and ${NQI}_{SQ}^{Act}$ is the Quality Index of the active sample related to the Sensory Quality. It is worth noting that each one of the normalized quality indices represents the percentage difference between the active sample (i.e., the sample fortified with the investigated by-product) and the control. The GQI was defined according to the following expression:

(14) $$GQI=\;\frac{\frac{1}{2}\cdot \left(\frac{{PQI}_{EI}^{CTR}-\;{PQI}_{EI}^{Act}}{{PQI}_{EI}^{CTR}}\cdot 100\right)\;+\;\frac{1}{2}\cdot \left(\frac{\frac{{{PQI}_{TDF}^{Act}\;-\;PQI}_{TDF}^{CTR}}{{PQI}_{TDF}^{CTR}}\cdot 100\;+\;\frac{{{PQI}_{ABTS}^{Act}\;-\;PQI}_{ABTS}^{CTR}}{{PQI}_{ABTS}^{CTR}}\cdot 100}{2}\right)}{\frac{{NQI}_{SQ}^{CTR}-\;{NQI}_{SQ}^{Act}}{{NQI}_{SQ}^{CTR}}\cdot 100}$$

### 2.10. Statistical Analysis

Statistical analysis was performed using JMP Student Edition 18 (SAS Institute Inc., Cary, NC, USA). One-way analysis of variance (ANOVA) was conducted, followed by Student’s *t*-test with the option for homogeneous groups (*p* < 0.05), to determine significant differences among the evaluated parameters.

## 3. Results and Discussion

### 3.1. Effect of Fortification on Sensory Quality of Fresh Pasta

**Figure 1:**
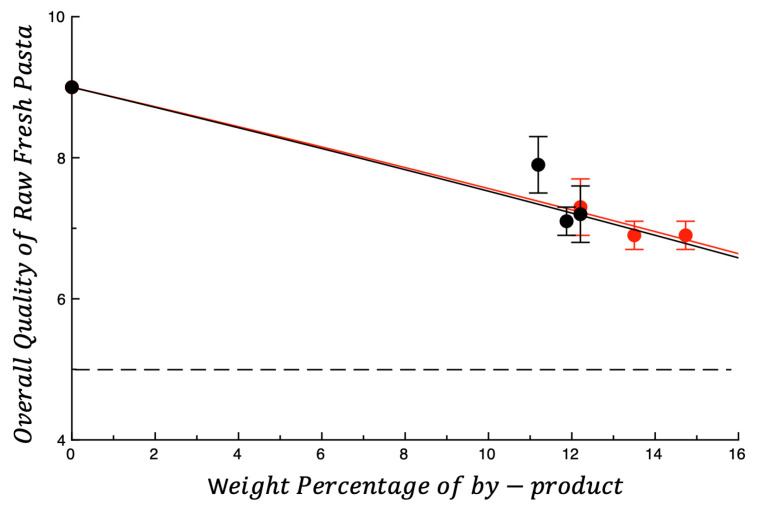
Overall quality of raw fresh pasta plotted as a function of the weight percentage of by-product. ● OP, ● AB. The lines shown in the figure are intended only to highlight data trends. Dotted line represents the threshold for acceptability (score = 5).

**Figure 2:**
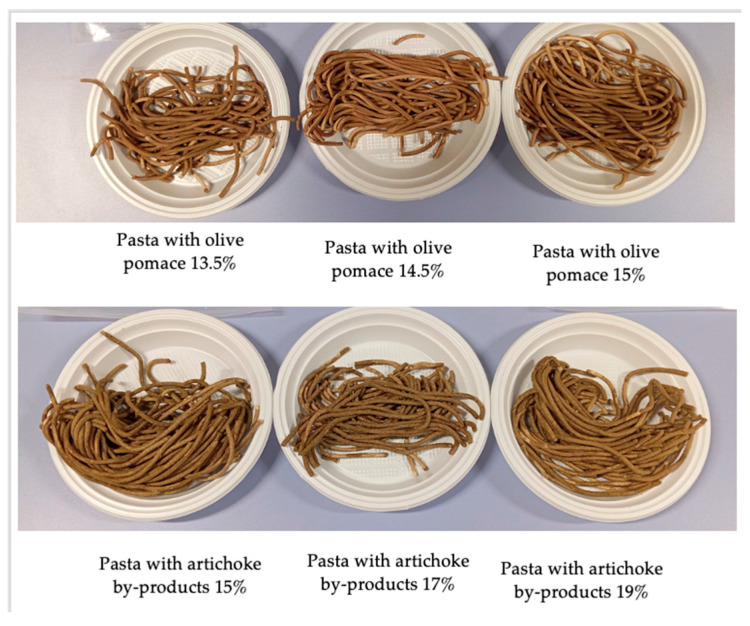
Photo of the six fortified raw pasta samples.

Figure 1 shows the overall quality of raw pasta plotted as a function of the weight percentage of the investigated by-products. The dashed horizontal line shown in the figure is the overall quality threshold, below which raw fresh pasta is considered unacceptable. As expected, the overall quality decreases as the weight percentage increases. In fact, the presence of the by-product worsens all sensory attributes, thus affecting the overall quality (Table S2); as expected, sensory attributes worsen as the concentration of by-product increases. To give an idea of raw pasta, the photo in Figure 2 below reports the six fortified samples. Previous studies also show that fibrous by-products disrupt dough uniformity and weaken the gluten network through physical and chemical interactions [50,51]. Soluble fibers (such as β-glucans, inulin, and psyllium) compete with starch for water and protein binding, which weaken the starch–protein interaction/bond and partially dehydrate the gluten [52]. In the current research, the attribute that most suffers from the presence of tested powders is the resistance to breaking. Most probably the by-product particles, interfering with the formation of the gluten network, reduce the resistance to breaking of raw pasta, which in turn affects the overall quality. Similar results have been reported for resistance to break of uncooked spaghetti with the addition of 15% olive pomace flour [53]. From what is shown in the figure, the type of by-product does not influence the way in which the overall quality of the raw pasta decreases with the by-product content.

**Figure 3:**
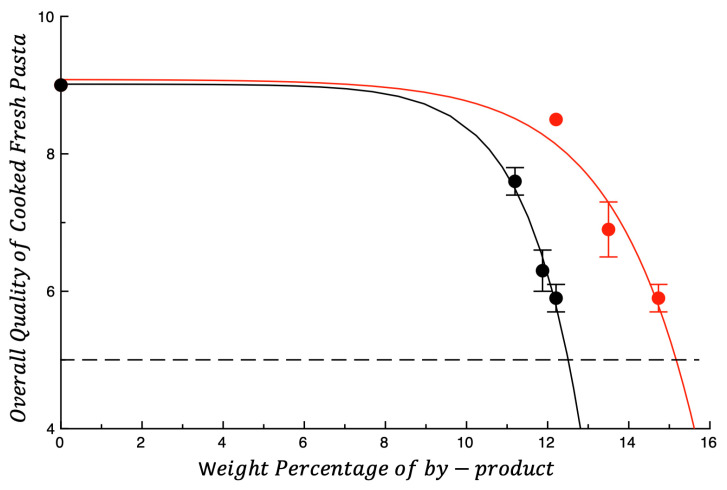
Overall quality of cooked fresh pasta plotted as a function of the weight percentage of by-product. ● OP = olive pomace; ● AB = artichoke by-products. The curves shown in the figure are intended to highlight data trends. Dotted line represents the threshold for acceptability (score = 5).

Figure 3 shows the overall quality of cooked fresh pasta plotted as function of the by-product weight percentage. The dashed horizontal line shown in the figure is the overall quality threshold. As observed for raw pasta, a reduction in the overall quality of cooked pasta is also observed as the concentration of by-product increases. However, unlike what was observed for raw *troccoli*, the type of by-product influences the way in which the overall quality of cooked pasta decreases with the by-product content (*p* < 0.05). Specifically, OP reduces overall quality faster than AB.

**Figure 4:**
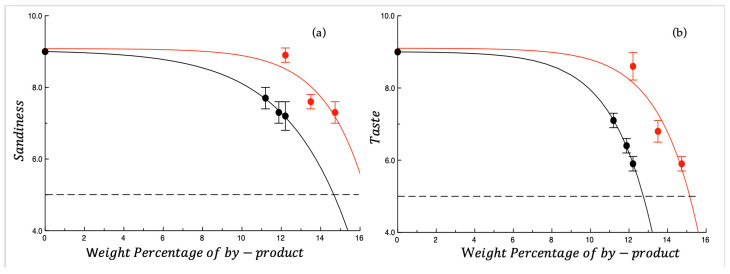
(**a**) Sandiness and (**b**) taste of cooked fresh pasta plotted as a function of the weight percentage of by-product. ● OP = olive pomace; ● AB = artichoke by-products. The curves shown in the figure are intended to highlight data trends. Dotted lines represent the threshold for acceptability (score = 5).

Sandiness and taste are the two sensory attributes that most influenced the overall quality of cooked pasta. Indeed, the way in which other sensory attributes decreased with the percentage of by-product did not depend on the type of by-product (Table S3). Figure 4a,b shows the sandiness and taste plotted as a function of the weight percentage of by-product. As can be observed in both graphs, the type of by-product significantly influences how the sensory attribute decreases as the by-product concentration increases. The presence of OP worsens the above sensory attributes more rapidly than AB. A decline in the taste was also observed in previous research where incorporation of olive by-products gave an intense taste to the food, which further compromised the sensory acceptability. This pungent taste might be due to the high content of polyphenols in OP [54,55].

**Figure 5:**
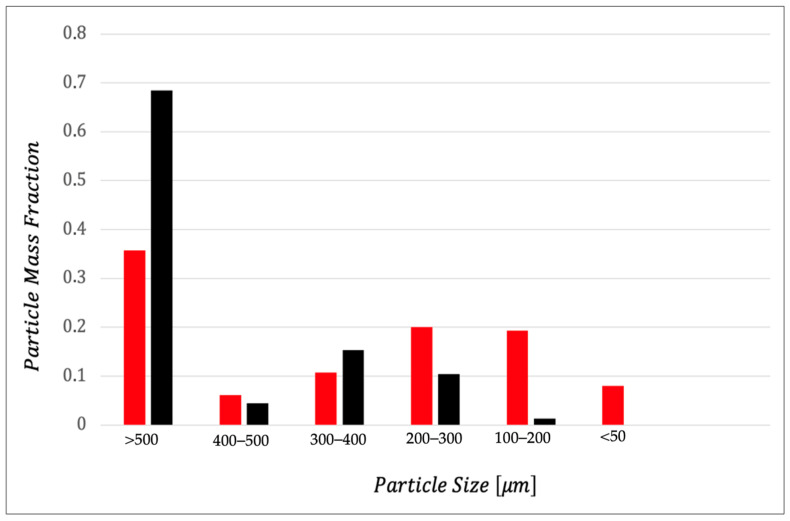
Particle size distribution. ■ OP = olive pomace; ■ AB = artichoke by-products.

Granulometry of powder significantly influences the rheological properties of dough, as well as the texture and sensory characteristics of final products [56]. Regarding the sandiness, Figure 5 shows the granulometric distribution of the two by-products studied. From what is shown in the figure, it is not surprising that OP worsens specific sensory attribute more markedly than AB. In fact, OP is richer in particles with a larger diameter than AB. Jahanbakhshi and Ansari [57] prepared sponge cake with olive stone powder and found that powder could be perceived as coarse particles during chewing. Another research group observed the interaction of olive pomace with regular flour and semolina and found that semolina, with its coarser texture, likely led to less effective mixing with dried OP, resulting in a granular pasta texture [58]. Dough prepared with wheat fiber demonstrated that small particle size significantly lowers water absorption than medium or large particles. Starch gelatinization was also influenced by particle size, with pasta containing medium and large bran particles showing higher gelatinization in the outer and intermediate regions compared to pasta with small particles [59].

### 3.2. Effect of Fortification on Technological Quality of Fresh Pasta

Table 2 shows the technological properties of the investigated samples. As one would expect, as the concentration of by-product increases, the pasta technological properties worsen, regardless of the type of by-product used for fortification. This circumstance is consistent with what was found for the sensory quality of the samples examined, and it further supports the idea that the by-product particles interfere with the formation of the gluten network. This aspect in turn leads to a deterioration of pasta structure and therefore compromises the technological properties. Several studies have reported similar outcomes regarding the enrichment of pasta with different by-products [60]. Addition of byproducts to pasta induces complex protein–dietary fiber–polyphenol interactions that have a significant impact on pasta quality and its cooking properties. Pasta enriched with dietary fiber, such as that fortified with grape pomace [61], exhibited a strong water-holding capacity as dietary fibers competed with gluten proteins for available moisture during dough formation, enhancing the water penetration rate and facilitating prior starch gelatinization [59]. On the other side, non-covalent interactions between polyphenols and gluten can lead to a denser gluten structure, facilitating intermolecular crosslinking and strengthening the gluten network. To this regard, Guo et al. [62] analyzed the effect of polyphenols, in the form of grape skin anthocyanin extract, on the microstructure and physicochemical properties of wheat gluten protein, demonstrating a better gluten structure. In the current work, for each technological parameter, an increase was observed as the concentration of by-product increased. However, within the same type of by-product, the observed increase is not statistically significant (*p* > 0.05). In fact, there are three groups of samples, the differences among which are statistically significant: CTRL, fortified samples with OP, and fortified samples with AB. Therefore, it is appropriate to make comparisons only between the two types of by-products, without specifying their concentration. Data listed in Table 2 show that AB worsens the technological properties more than OP. These results are in line with previous research showing that substituting semolina with 10% artichoke powder increases the water absorption of pasta. This effect may be attributed to the presence of fibers, particularly inulin, which are hydrophilic macromolecules. The higher cooking loss observed could be due to a weakened gluten network in the fortified dough, a finding consistent with earlier studies [63].

Some sensory attributes of cooked pasta, such as elasticity, firmness, and adhesiveness, are closely related to the technological properties of pasta. In fact, just as the technological properties decreased with increasing by-product concentration, the above-mentioned attributes also decreased (Table S3). However, unlike what was observed for the technological properties, no difference was noted in how the two types of by-products studied influenced the sensory attributes (*p* > 0.05). This observation can be attributed to the fact that panelists are unable to achieve the same level of sensitivity that can be obtained through the instrumental determination of technological properties.

### 3.3. Effect of Fortification on Nutritional Quality of Fresh Pasta

In Table 3 is reported the chemical quality of raw materials. As can be seen, both by-products present comparable content of total phenols and antioxidant activity (*p* > 0.05). Many times, these chemical parameters are highly correlated [64,65]. Total flavonoids are more abundant in AB (*p* < 0.05). This evidence does not surprise us considering other literature findings [66]. Dietary fiber data suggest that OP contains higher dietary fiber content compared to AB (*p* < 0.05). These results are consistent with previous studies, suggesting a similar trend in dietary fiber distribution [67,68]. However, concentrations of these bioactive compounds may vary based on factors such as plant species, particular plant section, and extraction technique applied.

In line with the results recorded in raw materials, Table 4 reports data on pasta chemical quality. As can be seen, the control pasta recorded a very low level of total phenols, total flavonoids, and antioxidant activity. The values for the control are statistically different (*p* < 0.05) from the other fortified samples. On the contrary, pasta enriched with by-products increased the nutritional level, even though the increase in by-products did not always promote an increased nutritional parameter. As a matter of fact, no statistically significant differences (*p* > 0.05) were found among by-product added samples in terms of total phenols. AB promoted a slight increase in total flavonoids compared to OP, and the same trend was recorded in terms of antioxidant activity (*p* > 0.05) (Figure S1).

Regarding the total dietary fiber content, the control sample showed the lowest total dietary fibers value (3.10 ± 0.18), which was significantly lower than all the fortified samples (*p* < 0.05). Among them, OP_Low, OP_Med, OP_High, AB_Med, and AB_High showed no statistically significant differences (*p* > 0.05). This indicates that increasing the level of by-product from medium to high did not show any significant variation within these groups (*p* > 0.05). However, the AB_Low sample was significantly lower than all other fortified samples but higher than the control (*p* < 0.05). This suggests that low-level addition of the artichoke by-product enhances fiber content compared to the control but may not reach the higher fiber levels as seen in medium or high additions. Moreover, fiber content in all enriched samples places these products within the category of foods eligible for the “high fiber” claim as it surpasses the 6% threshold, as defined by the nutritional criteria mentioned in the Annex of Regulation (EC) No. 1924/2006 [69]. As one would expect, as the by-product content increases, the fiber concentration in the fresh pasta increases. However, unlike what was observed for the cooked pasta overall quality, OP improved the nutritional quality of pasta more effectively than the AB (Figure S2). Baker et al. [70] reported that OP contains dietary fiber and phenolic compounds at concentrations of 620 and 4 g/kg dw, respectively, in a sieved fraction of the pomace. This suggests that OP is a rich source of dietary fiber, mainly in the form of insoluble dietary fiber. Fortification of pasta with 5% and 10% OP showed to increase dietary fiber by approximately 83% and 166%, respectively [23]. Another study used grape pomace and olive pomace pâté to fortify tagliatelle, and the results showed an approximate 3% increase in fiber content [71].

### 3.4. Energy Consumption of By-Product Dehydration Process

**Figure 6:**
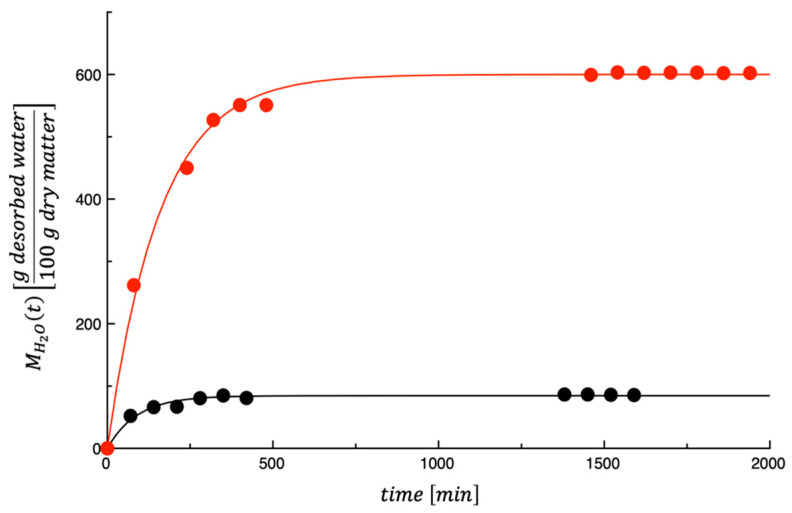
Dehydration kinetics of the investigated by-products. ● OP = olive pomace; ● AB = artichoke by-products.

Figure 6 shows the dehydration kinetics of the investigated by-products. The curves shown in the figure are the best fit of the model proposed by Conte et al. [32] (i.e., Equations (4) and (5)) to the dehydration kinetic data. As shown in the figure, there is a substantial difference between the dehydration kinetics of the two by-products. This is because the initial water content of the two by-products is very different from each other. In particular, the initial value of water mass fraction (i.e., ${1-x}_{dm}^{0}$) for OP and AB are 0.4717 ± 0.008054 and 0.8537 ± 0.02970, respectively.

The mean relative deviation modulus $\left(\bar{E}\%\right)$ was used to measure the goodness of fit [72]:

(15) $$\bar{E}\%\;=\;\frac{100}{N}\cdot \sum_{i=1}^{i=N}\frac{\left|M_{i}^{exp}-\;M_{i}^{pred}\right|}{M_{i}^{exp}}$$

where N is the number of experimental data, $M_{i}^{exp}$ is the experimental value, and $M_{i}^{pred}$ is the predicted value.

The values of the model’s parameter calculated by fitting the dehydration kinetics along with the computed values of $\bar{E}\%$ are shown in Table 5. As shown by the $\bar{E}\%\;$ values, the model proposed by Conte et al. [32] satisfactorily describes the dehydration kinetic of the investigated by-products.

Figure S3 shows the energy consumed by the dehydrator ($E\left(t\right)$) plotted as a function of time. The curves shown in the same figure are the best fit of a straight line through the origin of the data. The calculated values of the slope of the straight line (i.e., γ) are shown in Table 5 along with the computed value of $\bar{E}\%$. Concerning this latter value, the values obtained indicate that a straight line through the origin adequately describes the data, thus implying that the power supplied to the dehydration chamber (i.e., γ) can be acceptably considered constant during the entire process. Considering the computed γ, the differences between the two samples are small, such that they can be considered negligible.

**Figure 7:**
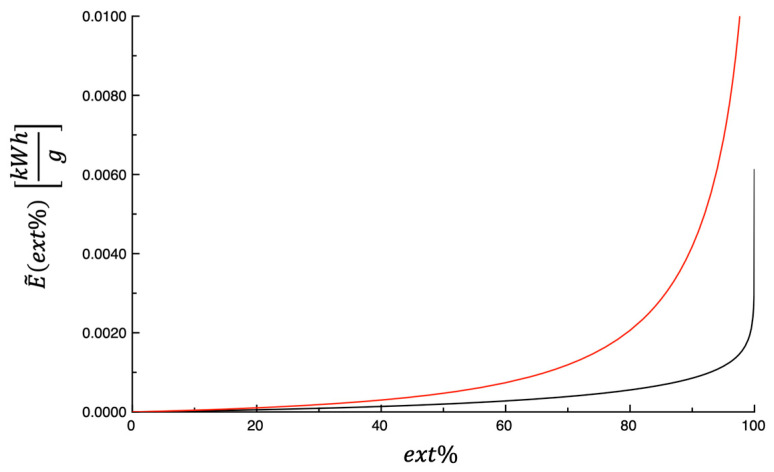
Energy consumption per gram of dehydrated food ($\tilde{E}\left(ext\%\right)$) plotted as a function of ext%. 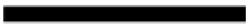 OP = olive pomace; 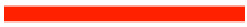 AB = artichoke by-products.

Figure 7 shows $\tilde{E}\left(ext\%\right)$ plotted as a function of ext%. The curves shown in the figure were predicted by means of Equations (6) and (7), whereas the values of the model parameters used are those listed in Table 5. The amount of energy consumed per gram of dehydrating by-product was calculated by setting the extent of the dehydration process at 99% (i.e., $\tilde{E}\left(99\%\right)$); the obtained values are shown in Table 5. As can be observed from data shown in the table, there is a marked difference between the investigated by-products, which are of an order of magnitude. Considering that the power supplied to the dehydrator during the process does not depend on the type of by-product, the strong difference found in $\tilde{E}\left(99\%\right)$ for the two by-products might seem anomalous. In fact, the initial content of water differs greatly between OP and AB. This involves a significant difference in the time needed to reach a degree of dehydration equal to 99%, which requests a significant increase in energy necessary for dehydration.

Figure S4 shows the time needed to reach a given extent of the dehydration process (i.e., $t_{ext\%}$), plotted as a function of ext%. As expected, the curve for AB is always above that for OP. This means that whatever the level of dehydration of the by-product, the time needed to reach that level will always be greater for AB than for OP. In fact, $t_{99\%}$ for AB and OP is 729.97 min and 414.73 min, respectively. This explains the marked difference observed before between the $\tilde{E}\left(99\%\right)$ values.

### 3.5. Environmental Impact

**Figure 8:**
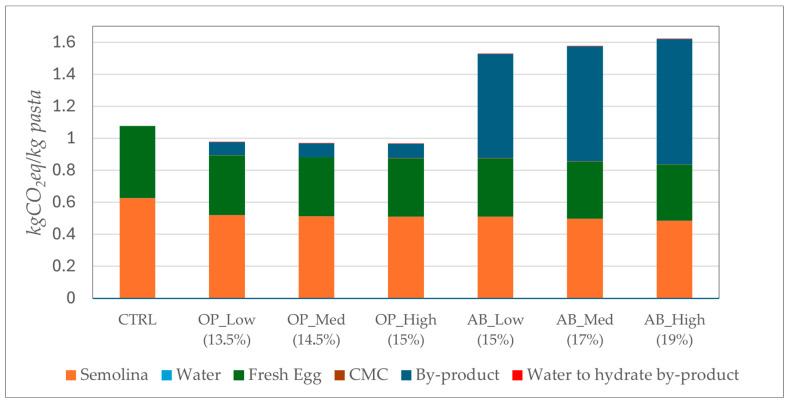
Carbon footprint of pasta samples, expressed in kgCO_2_eq per kg of pasta. Color coding is used to indicate the contribution of each ingredient.

In this section, the results concerning the environmental impact in terms of carbon footprint are presented for the control pasta and the variants fortified with the two different types of by-products. Figure 8 illustrates the carbon footprint associated with the production of 1 kg of each pasta sample. As shown, the pasta fortified with AB exhibits the highest values exceeding those of both control sample and other fortified variants. The comparison between the control sample and the three pasta samples fortified with AB reveals higher carbon footprint values for the latter, primarily due to the energy consumption associated with the processing of artichoke by-products, despite the reduced use of semolina, water, and fresh eggs.

Indeed, the carbon footprint values of the AB_Low (15%), AB_Med (17%), and AB_High (19%) are 1.53 kgCO_2_eq, 1.58 kgCO_2_eq, and 1.62 kgCO_2_eq, respectively, and are higher than that of the control sample, which amounts to 1.08 kgCO_2_eq. The energy demand associated with the processing of AB results to be more than seven times higher than the energy required for processing OP. This is reflected in the lower carbon footprint values observed for the OP_Low (13.5%), OP_Med (14.5%), and OP_High (15%), which amount to 0.98 kgCO_2_eq, 0.97 kgCO_2_eq, and 0.96 kgCO_2_eq, respectively. Moreover, these values are slightly lower than those of the control sample (1.08 kgCO_2_eq), primarily due to the reduced quantities of semolina, water, and fresh eggs used in the pasta formulation, as well as the lower energy input required for processing OP.

### 3.6. Global Quality Index

The Global Quality Index (GQI), as delineated by Lordi et al. [49], was employed to identify the sample that most effectively balances the positive and negative attributes associated with by-product enrichment. The GQI provides a comprehensive evaluative metric that integrates multiple quality parameters into a single quantitative value, thereby enabling an objective comparison among samples. Its application facilitates the selection of the sample that optimizes beneficial characteristics, such as nutritional enhancement and environmental impact, while minimizing adverse effects, such as sensory deterioration. This methodological approach ensures a rigorous and systematic assessment of sample quality in the context of enrichment strategies. As reported in the work of Lordi et al. [49], the numerator of Equation (14) represents the arithmetic mean of the environmental impact and the nutritional quality. The nutritional quality component is calculated as the arithmetic mean of the normalized quality indices associated with the nutritional attributes of fortified pasta. The denominator corresponds to the normalized quality index related to sensory quality. A GQI value greater than 1 indicates that the positive effects of fortification outweigh the negative aspects, whereas a GQI less than 1 suggests the opposite. Consequently, the sample with the highest GQI is considered to have the optimal overall performance, as it most effectively balances the beneficial and adverse effects associated with fortification.

**Figure 9:**
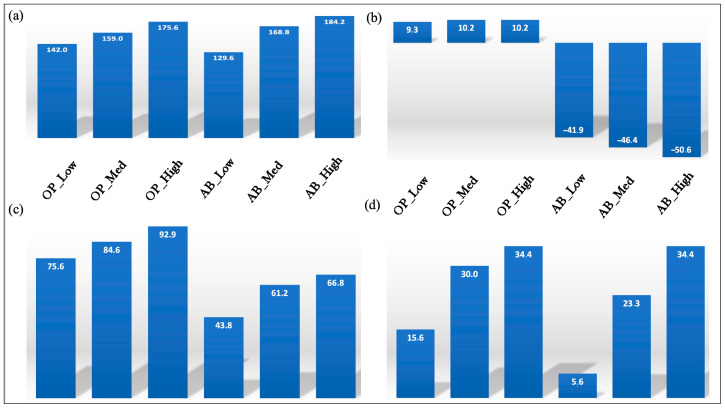
(**a**) The arithmetic means of the normalized quality indices associated with the nutritional quality of fortified fresh pasta; (**b**) the normalized quality index associated with the environmental impact of the fortified fresh pasta; (**c**) the arithmetic means of the normalized quality indices associated with the nutritional quality and environmental impact of the fortified fresh pasta; (**d**) the normalized quality index associated with the sensory quality of the investigated fortified pasta with olive pomace (OP) and artichoke by-products (AB).

In Figure 9a the arithmetical means of the normalized quality indices associated with nutritional quality of the fortified fresh pasta are shown. Based on the information presented in the figure, it can be inferred that as the amount of by-product increases, the nutritional quality of the fortified pasta also improves. This result is consistent with the findings discussed earlier. It is interesting to note that the average percentage increase in nutritional quality exceeds 142 across all samples fortified with OP, whereas, in the case of fortification with AB, the sample with the highest concentration of by-product shows a percentage increase slightly below 200%. What was observed is a direct consequence of the nutritional characteristics of the investigated by-products.

In Figure 9b, the normalized quality index associated with the environmental impact of the investigated samples is shown. As can be observed from the figure, in the case of pasta fortified with OP, a benefit is achieved through the reuse of the by-product. However, it is important to note that the environmental advantage is minimal when compared to the benefit associated with the enhancement of the nutritional quality of the fortified pasta with the same by-product. When considering the pasta fortified with AB, an increase in environmental impact compared to the control is observed. As previously discussed, this result can be attributed to the energy consumption associated with the dehydration of AB. It is worth noting that the extent of the worsening in environmental impact is still less significant than the improvement achieved in terms of nutritional quality.

In Figure 9c, the arithmetic means of the normalized quality index associated with the environmental impact and nutritional quality are shown. As expected, the pasta fortified with OP exhibits higher values compared to that fortified with AB. Furthermore, the values tend to increase with the increasing concentration of the by-product. It is noteworthy that, despite the less favorable environmental impact results associated with fortification, particularly in the case of AB, which even shows negative values, the observed values are still significantly greater than 1. Figure 9d shows the normalized quality index associated with the sensory quality of the investigated fortified pasta. As expected, the sensory quality declines with increasing concentrations of the by-product. Additionally, OP has a greater negative impact on sensory quality compared to AB.

**Figure 10:**
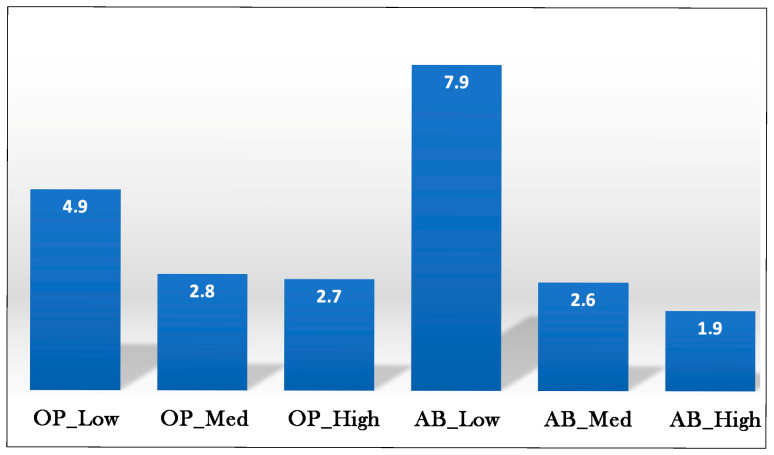
The GQI of fresh pasta with olive pomace (OP) and artichoke by-products (AB).

In Figure 10 the GQI of the investigated samples is shown. As can be observed from data presented in this figure, all values are greater than 1. This indicates that the benefits associated with fortification outweigh the disadvantages. For both by-products used in pasta fortification, a decrease in the GQI is observed as the concentration of the by-product increases. Indeed, the same trend is observed for the decline in sensory quality, which decreases as the concentration of by-product increases. This indicates that, in determining the trend of the GQI with respect to the by-product concentration, the denominator of Equation (14) (i.e., sensory quality) has a greater influence than the numerator (i.e., the arithmetic mean of environmental impact and nutritional quality). Among the investigated samples, pasta with the highest GQI is the sample with the lowest by-product concentration, and between the two investigated by-products, AB is the one with the highest GQI. This is because, at its lowest concentration, AB has the least detrimental impact on the sensory quality of pasta.

## 4. Conclusions

A comparison among pasta fortified with three different concentrations of two by-products was made experimentally. Sensory quality of control and fortified pasta samples revealed that by-product addition worsens pasta acceptance, and the effects are more marked as the concentration of by-product increases. Regardless, pasta with both by-products remained above the threshold (score = 5) also when the highest concentrations were added. From a technological point of view, both olive pomace and artichoke by-products slightly compromised pasta cooking quality because it is common that by-product particles interfere with the gluten network, thus weakening the structure. Regardless, the differences with the control revealed that fortified pasta samples have an acceptable technological quality. The nutritional content of fortified pasta was enhanced. In terms of total phenols, flavonoids, antioxidant activity, and fibers, all the by-product-added samples were found richer than the control. The chemical analyses on the two raw materials justified results recorded in the fortified samples. The environmental impact analysis demonstrated that the carbon footprint of the pasta fortified with artichoke by-products exhibited the highest values, thus exceeding those of control and olive pomace-fortified variants. To calculate the GQI, technological quality, nutritional properties, environmental impact, and sensory quality were properly combined. Results highlighted that a balance among these indices was found, and it could be used to estimate the suitability of by-product recycling. As a matter of fact, comparing the GQI values, the most interesting solution comes from pasta with the 15% artichoke by-product, with this sample being very interesting from a nutritional point of view and more acceptable for sensory quality. These aspects therefore balanced the drawback linked to a negative environmental impact index, due to the energy necessary to process the by-products. Therefore, the findings emphasize the crucial role of improving energy efficiency during by-product processing to achieve significant environmental benefits. Adopting effective strategies for the management and valorization of by-products represents a key element for minimizing environmental impacts and fostering the development of a sustainable bioeconomy within industrial sectors. Although there are potential applications for the valorization and exploitation of food agro-industrial by-products, they have not been implemented on a big scale nor in a real waste management case, which could lead to the generation of new business [73]. Key challenges include the need for efficient, scalable extraction and conversion technologies. Opportunities lie in establishing circular bioeconomy models that integrate fruit waste valorization into various industries, creating value from waste and promoting sustainability. At the research level, future work should be carried out to verify that bioactive compounds recorded in fortified samples remain stable after cooking and digestion. Considering that the presence of active molecules could also promote a better maintenance of pasta quality during refrigerated storage, another topic to be investigated could be the effects of bioactive compounds on pasta shelf life.

## Figures and Tables

**Table 1:** Ingredients of control and fortified fresh pasta samples.

Samples	Semolina (g)	Distilled Water (g)	Fresh Egg (g)	CMC (g)	By-Product (g)	Water to Hydrate By-Product (g)
CTRL	1035	270	195	0	0	0
AB_Med	1035	270	195	0.77	225	117
AB_Low	1035	270	195	0.80	255	133
AB_High	1035	270	195	0.83	285	148
OP_Low	1035	270	195	0.75	202	105
OP_Med	1035	270	195	0.76	217	113
OP_High	1035	270	195	0.77	225	117

CTRL = Control Pasta. AB_Low (15%), AB_Med (17%), AB_High (19%) = artichoke by-products; OP_Low (13.5%), OP_Med (14.5%), OP_High (15%) = olive pomace.

**Table 2:** Technological properties of control and fortified fresh pasta.

Samples	OCT (min)	Cooking Loss (%)	Swelling Index (g Water/g Dry Pasta)	Water Absorption (%)
AB_High	13	4.54 ± 0.11 ^a^	1.84 ± 0.01 ^a^	65.47 ± 1.47 ^a^
AB_Med	12	4.33 ± 0.15 ^a^	1.82 ± 0.01 ^a^	63.05 ± 2.14 ^a^
AB_Low	11.30	4.32 ± 0.13 ^a^	1.81 ± 0.01 ^a^	59.87 ± 1.80 ^a,b^
OP_High	11.30	3.26 ± 0.09 ^b^	1.65 ± 0.09 ^b^	53.72 ± 4.23 ^b,c^
OP_Med	11.30	3.09 ± 0.05 ^b^	1.64 ± 0.08 ^b^	52.31 ± 0.91 ^c^
OP_Low	11	3.03 ± 0.24 ^b^	1.62 ± 0.01 ^b^	51.63 ± 0.47 ^c^
CTRL	9.30	2.37 ± 0.12 ^c^	1.55 ± 0.03 ^b^	50.44 ± 3.97 ^c^

CTRL = control Pasta; AB_Low (15%), AB_Med (17%), AB_High (19%) = artichoke by-products; OP_Low (13.5%), OP_Med (14.5%), OP_High (15%) = olive pomace. Data in each column with different superscript lowercase letters (a–c) show significant differences between pasta samples (*p* < 0.05).

**Table 3:** Total phenols (mg GAE/g dw), total flavonoids (mg QE/g dw), antioxidant activity (mg TE/g dw), and total dietary fibers (g/100 g) of each by-product.

Powder Samples	Total Phenols (mg GAE/g dw)	Total Flavonoids (mg QE/g dw)	Antioxidant Activity (mg TE/g dw)	TDF (g/100 g)
OP	38.38 ± 5.56 ^a^	35.64 ± 2.77 ^b^	30.48 ± 6.68 ^a^	59.6 ± 3.4 ^a^
AB	36.88 ± 3.50 ^a^	54.69 ± 4.25 ^a^	31.07 ± 3.01 ^a^	43.0 ± 2.4 ^b^

Data are presented as mean ± SD (n = 3). Data in each column with different superscript lowercase letters (a,b) show significant differences between powder samples (*p* < 0.05). GAE = gallic acid equivalents; QE = quercetin equivalents; TE = Trolox equivalents; TDF = total dietary fibers; OP = olive pomace; AB = artichoke by-products.

**Table 4:** Total phenols (mg GAE/g dw), total flavonoids (mg QE/g dw), antioxidant activity (mg TE/g dw) and total dietary fibers (TDF, g/100 g) of *troccoli* samples.

Samples	Total Phenols (mg GAE/g dw)	Total Flavonoids (mg QE/g dw)	Antioxidant Activity (mg TE/g dw)	TDF (g/100 g)
CTRL	1.76 ± 0.08 ^b^	1.39 ± 0.13 ^c^	0.88 ± 0.06 ^d^	3.10 ± 0.18 ^c^
OP_Low	2.76 ± 0.24 ^a^	2.07 ± 0.30 ^b^	1.20 ± 0.16 ^c^	10.60 ± 0.61 ^a^
OP_Med	2.99 ± 0.11 ^a^	2.57 ± 0.13 ^a,b^	1.24 ± 0.07 ^c^	11.50 ± 0.66 ^a^
OP_High	3.03 ± 0.03 ^a^	2.58 ± 0.20 ^a,b^	1.36 ± 0.07 ^c^	12.10± 0.70 ^a^
AB_Low	3.01 ± 0.25 ^a^	2.64 ± 0.50 ^a^	1.56 ± 0.08 ^b^	8.60 ± 0.50 ^b^
AB_Med	3.06 ± 0.28 ^a^	2.75 ± 0.32 ^a^	1.73 ± 0.08 ^a^	10.40 ± 0.60 ^a^
AB_High	3.17 ± 0.46 ^a^	2.80 ± 0.33 ^a^	1.77 ± 0.05 ^a^	11.20 ± 0.65 ^a^

Data are presented as mean ± SD (n = 3). Data in each column with different superscript lowercase letters (a–d) show significant differences among *troccoli* samples (*p* < 0.05). GAE = gallic acid equivalents; QE = quercetin equivalents; TE = Trolox equivalents; TDF = total dietary fibers; CTRL = control sample; AB_Low (15%), AB_Med (17%), AB_High (19%) = artichoke by-products; OP_Low (13.5%), OP_Med (14.5%), OP_High (15%) = olive pomace.

**Table 5:** Parameters used to estimate dehydration kinetics and energy consumption.

Parameters	OP	AB
$t_{c}\left[min\right]$	3.41 × 10^−6^	8.89 × 10^−6^
$k_{1}\left[\frac{g\;desorbed\;water}{100\;g\;dry\;matter}\cdot \frac{1}{min}\right]$	0.938	3.79
$k_{2}\;\left[\frac{g\;desorbed\;water}{100\;gdry\;matter}\right]$	84.45	599.97
$\gamma \;\left[\frac{kWh}{min}\right]$	1.49 × 10^−2^	1.44 × 10^−2^
$x_{dm}^{0}$	5.28 × 10^−1^	1.46 × 10^−1^
$m_{Tot}^{0}\;\left[g\right]$	6000	6000
$\bar{E}\%$ Dehydration Kinetic	4.11	1.66
$\bar{E}\%\;$ Energy Consumption	3.14	1.73
$\tilde{E}\left(99\%\right)\left[\frac{kWh}{g}\right]$	1.83 × 10^−3^	1.34 × 10^−2^

## Data Availability

The original contributions presented in the study are included in the article/Supplementary Materials. Further inquiries can be directed to the corresponding authors.

## Supplementary Materials

The following supporting information can be downloaded at: https://www.mdpi.com/article/10.3390/foods14193379/s1, Table S1: Ingredients used in the pasta preparation and corresponding source of environmental impact data; Table S2: Values of sensory parameters of raw fresh pasta with olive pomace (OP) and artichoke by-products (AB) and relative control sample; Table S3: Values of sensory parameters of cooked fresh pasta with olive pomace (OP) and artichoke by-products (AB) and relative control sample; Figure S1: ABTS of fresh pasta plotted as a function of the weight percentage of by-product; Figure S2: Total dietary fiber content plotted as function of the weight percentage of by-product; Figure S3: Amount of energy supplied to the dehydrator (*E*(*t*)) plotted as a function of time; Figure S4: Time needed to reach a given extent of dehydration, plotted as a function of *ext*%.
