# Supplementary material for: Assessment of Quality and Environmental Impact of Artisanal Fresh Pasta Fortified with Agri-Food By-Products
Source: Foods. 2025 Sep 29;14(19):3379. doi: 10.3390/foods14193379 (PMC12523257; doi:10.3390/foods14193379)
Supplement: Supplementary file 1 [file foods-14-03379-s001.zip › foods-3868889-supplementary.pdf]

Supporting Data for the Article

Assessment of Quality and Environmental Impact of Artisanal Fresh Pasta Fortified with Agri-Food By-Products

Table S1. Ingredients used in the pasta preparation and corresponding source of environmental impact data.

Ingredient	Value CTRL	Value OP_Low(13.5%)	Value OP_Med (14.5%)	Value OP_High(15%)	Value AB_Low(15%)	Value AB_Med(17%)	Value AB_High(19%)	Unit	Source	Name of the data used in the database	Reference
**Semolin**	690	572.17	565.03	561.53	561.53	547.95	535.01	g/kg	University of Foggia		Environdec, (2022)[44]
**Water**	180	149.26	147.40	146.48	146.48	142.94	139.57	g/kg	University of Foggia	Tap water {Europe without Switzerland}| tap water production, conventional treatment | Cut-off, S	Ecoinvent (2024)[47]
**Fresh Egg**	130	107.80	106.45	105.80	105.79	103.23	100.80	g/kg	University of Foggia		Guillaume et al.,2022; Leinonen et al., 2012.[45, 46]
**CMC**	0	0.415	0.418	0.42	0.42	0.426	0.432	g/kg	University of Foggia	Carboxymethyl cellulose, powdered {RoW} production | Cut-off, S	Ecoinvent (2024)

<table>
<tr><td>By-product</td><td>0</td><td>111.95</td><td>118.74</td><td>122.07</td><td>122.07</td><td>135</td><td>147.32</td><td>kWh/kg</td><td>University of Foggia</td><td>Electricity, medium voltage {IT}|market for | Cut-off, S</td><td>Ecoinvent (2024)</td></tr>
<tr><td>Water to hydrate by-</td><td>0</td><td>58.40</td><td>61.95</td><td>63.69</td><td>63.69</td><td>70.44</td><td>76.86</td><td>g/kg</td><td>University of Foggia</td><td>Tap water {Europe without Switzerland}| tap water production, conventional treatment | Cut-off, S</td><td>Ecoinvent (2024)</td></tr>
</table>

Table S2. Values of sensory parameters of raw fresh pasta with olive pomace (OP) and artichoke by-products (AB) and relative control sample.

Raw OP samples	Color	Odor	Homogeneity	Appearance	Breaking strength	Overall quality
OP_High	8.71 ± 0.27^{a}	9.00 ± 0.00^{a}	9.00 ± 0.00^{a}	9.00 ± 0.00^{a}	$6.43 \pm 0.53^{b,\,c}$	7.21 ± 0.39^{c}
OP_Med	7.90 ± 0.20^{b}	8.08 ± 0.20^{b}	7.67 ± 0.26^{b}	8.50 ± 0.00^{b}	6.25 ± 0.27^{c}	7.08 ± 0.20^{c}
OP_Low	9.00 ± 0.00^{a}	9.00 ± 0.00a	9.00 ± 0.00^{a}	8.14 ± 0.24^{c}	7.00 ± 0.00^{b}	7.86 ± 0.38^{b}
CTRL	9.00 ± 0.00^{a}	9.00 ± 0.00^{a}	9.00 ± 0.00^{a}	9.00 ± 0.00^{a}	9.00 ± 0.00^{a}	9.00 ± 0.00^{a}
**Raw AB samples**	**Color**	**Odor**	**Homogeneity**	**Appearance**	**Breaking strength**	**Overall quality**
AB_High	8.00 ± 0.00^{b}	8.21 ± 0.27^{b}	7.93 ± 0.19^{c}	8.00 ± 0.01^{b}	6.21 ± 0.27^{b}	6.93 ± 0.19^{b}
AB_Med	8.07 ± 0.19^{b}	8.29 ± 0.27^{b}	7.93 ± 0.19^{c}	8.00 ± 0.01^{b}	6.43 ± 0.19^{b}	6.93 ± 0.19^{b}
AB_Low	9.00 ± 0.00^{a}	9.00 ± 0.00^{a}	8.43 ± 0.19^{b}	9.00 ± 0.00^{a}	5.57 ± 0.35^{c}	7.29 ± 0.39^{b}
CTRL	9.00 ± 0.00^{a}	9.00 ± 0.00^{a}	9.00 ± 0.00^{a}	9.00 ± 0.00^{a}	9.00 ± 0.00^{a}	9.00 ± 0.00^{a}

Data in each column with different superscript lowercase letters show significant differences between powder samples ($p < 0.05$).

Table S3. Values of sensory parameters of cooked fresh pasta with olive pomace (OP) and artichoke by-products (AB) and relative control sample.

Raw OP samples	Color	Odor	Elasticity	Bulkiness	Adhesiveness	Firmness	Sandiness	Taste	Overall quality
OP_High	$8.64 \pm 0.24^{b,c}$	8.64 ± 0.24^{a}	8.64 ± 0.24^{a}	8.50 ± 0.00^{b}	8.79 ± 0.27^{a}	$8.64 \pm 0.38^{a,b}$	7.21 ± 0.39^{b}	5.86 ± 0.24^{d}	5.86 ± 0.24^{d}
OP_Med	8.58 ± 0.20^{c}	8.67 ± 0.26^{a}	7.67 ± 0.26^{b}	8.50 ± 0.00^{b}	8.33 ± 0.26^{b}	8.25 ± 0.27^{b}	7.25 ± 0.27^{b}	6.42 ± 0.20^{c}	6.33 ± 0.26^{c}
OP_Low	9.00 ± 0.00^{a}	9.00 ± 0.00^{a}	8.64 ± 0.24^{a}	8.64 ± 0.24^{b}	$8.57 \pm 0.19^{a,b}$	$8.71 \pm 0.27^{a,b}$	7.71 ± 0.27^{b}	7.14 ± 0.24^{b}	7.64 ± 0.24^{b}
CTRL	9.00 ± 0.00^{a}	9.00 ± 0.00^{a}	9.00 ± 0.00^{a}	9.00 ± 0.00^{a}	9.00 ± 0.00^{a}	9.00 ± 0.00^{a}	9.00 ± 0.00^{a}	9.00 ± 0.00^{a}	9.00 ± 0.00^{a}
**Raw AB samples**	**Color**	**Odor**	**Elasticity**	**Bulkiness**	**Adhesiveness**	**Firmness**	**Sandiness**	**Taste**	**Overall quality**
AB_High	8.00 ± 0.01^{b}	7.79 ± 0.39^{b}	7.14 ± 0.24^{c}	8.00 ± 0.00^{b}	8.00 ± 0.01^{c}	7.50 ± 0.01^{c}	7.29 ± 0.27^{b}	5.93 ± 0.19^{c}	5.93 ± 0.19^{d}
AB_Med	8.00 ± 0.01^{b}	7.86 ± 0.24^{b}	7.64 ± 0.24^{b}	8.00 ± 0.00^{b}	8.00 ± 0.00^{c}	7.50 ± 0.00^{c}	7.57 ± 0.19^{b}	6.79 ± 0.27^{b}	6.86 ± 0.38^{c}
AB_Low	9.00 ± 0.00^{a}	9.00 ± 0.00^{a}	8.57 ± 0.19^{a}	8.93 ± 0.19^{a}	8.50 ± 0.01^{b}	8.50 ± 0.01^{b}	8.86 ± 0.24^{a}	8.64 ± 0.38^{a}	8.50 ± 0.00^{b}
CTRL	9.00 ± 0.00^{a}	9.00 ± 0.00^{a}	9.00 ± 0.00^{a}	9.00 ± 0.00^{a}	9.00 ± 0.00^{a}	9.00 ± 0.00^{a}	9.00 ± 0.00^{a}	9.00 ± 0.00^{a}	9.00 ± 0.00^{a}

Data in each column with different superscript lowercase letters show significant differences between powder samples ($p < 0.05$).

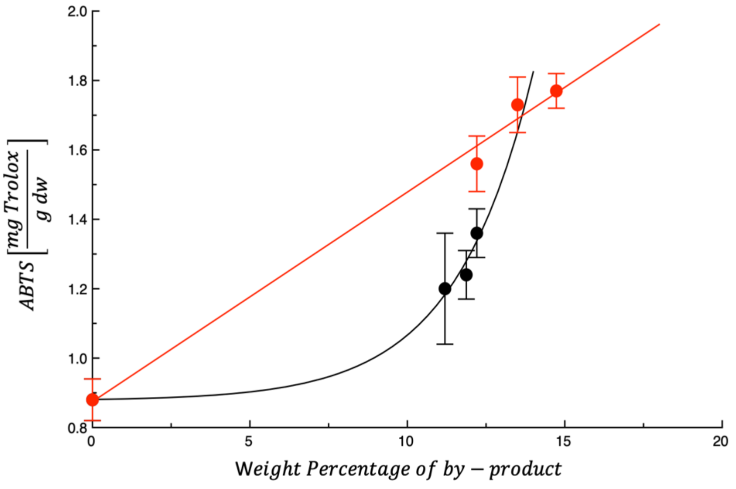

Figure S1: ABTS of fresh pasta plotted as a function of the weight percentage of by-product. ● OP = Olive pomace; ● AB = artichoke by-products. The curves shown in the figure are intended to highlight data trend.

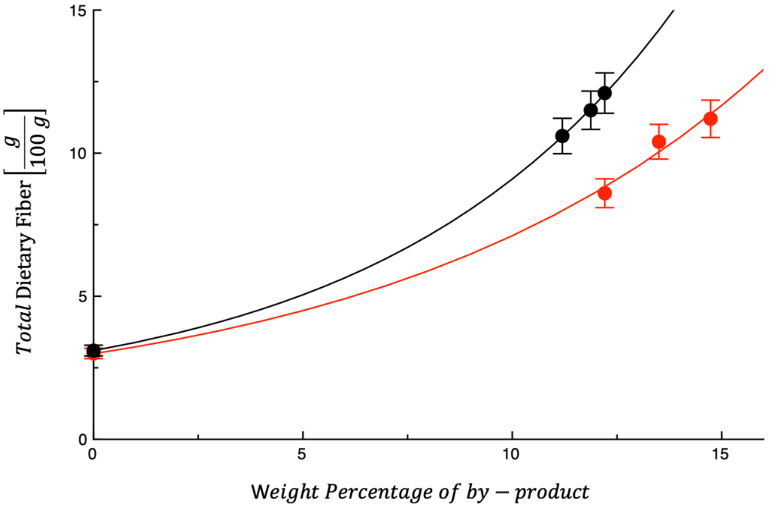

Figure S2: Total dietary fiber content plotted as function of the weight percentage of by-product. ● OP = Olive pomace; ● AB = artichoke by-products. The curves shown in the figure are intended to highlight data trend.

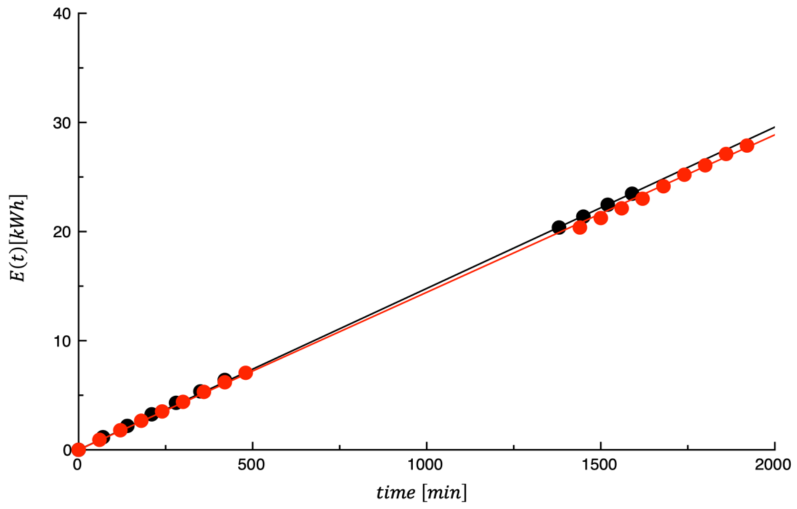

Figure S3: Amount of energy supplied to the dehydrator ($E(t)$) plotted as a function of time.

- OP = Olive pomace; ● AB = artichoke by-products.

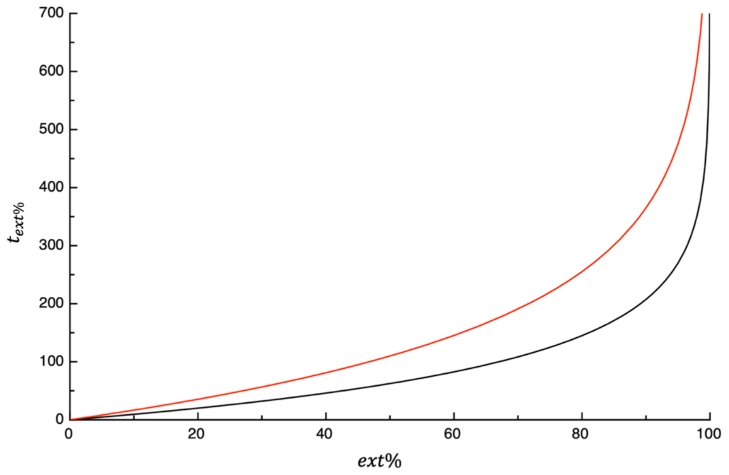

Figure S4: Time needed to reach a given extent of dehydration, plotted as a function of $ext\%$.
▬ OP = Olive pomace, ▬ AB = artichoke by-products.

References

47. Ecoinvent, 2024. Ecoinvent database version 3.

46. Guillaume, A. Hubatová-Vacková, V. Kočí, Environmental impacts of egg production from a life cycle perspective, Agric. 12(3) (2022) 355.

45. Leinonen, A.G. Williams, J. Wiseman, J. Guy, I. Kyriazakis, Predicting the environmental impacts of chicken systems in the United Kingdom through a life cycle assessment: Broiler production systems. Poultry Sci. 91(1) (2012) 8-25.

44. Environdec, 2022. Environmental Product Declaration: Pasta di Semola Agnesi. https://api.environdec.com/api/v1/EPDLibrary/Files/f53689a9-89dc-405c-9b45 08da3d5507cb/Data
